# High-Throughput Sequencing Reveals Diverse Sets of Conserved, Nonconserved, and Species-Specific miRNAs in Jute

**DOI:** 10.1155/2015/125048

**Published:** 2015-03-16

**Authors:** Md. Tariqul Islam, Ahlan Sabah Ferdous, Rifat Ara Najnin, Suprovath Kumar Sarker, Haseena Khan

**Affiliations:** Molecular Biology Laboratory, Department of Biochemistry and Molecular Biology, University of Dhaka, Dhaka 1000, Bangladesh

## Abstract

MicroRNAs play a pivotal role in regulating a broad range of biological processes, acting by cleaving mRNAs or by translational repression. A group of plant microRNAs are evolutionarily conserved; however, others are expressed in a species-specific manner. Jute is an agroeconomically important fibre crop; nonetheless, no practical information is available for microRNAs in jute to date. In this study, Illumina sequencing revealed a total of 227 known microRNAs and 17 potential novel microRNA candidates in jute, of which 164 belong to 23 conserved families and the remaining 63 belong to 58 nonconserved families. Among a total of 81 identified microRNA families, 116 potential target genes were predicted for 39 families and 11 targets were predicted for 4 among the 17 identified novel microRNAs. For understanding better the functions of microRNAs, target genes were analyzed by Gene Ontology and their pathways illustrated by KEGG pathway analyses. The presence of microRNAs identified in jute was validated by stem-loop RT-PCR followed by end point PCR and qPCR for randomly selected 20 known and novel microRNAs. This study exhaustively identifies microRNAs and their target genes in jute which will ultimately pave the way for understanding their role in this crop and other crops.

## 1. Introduction

Regulation of gene expression is one of the most enigmatic facets of molecular genetics that results in intricate appearance of a biological entity. Scientists have been attempting to elucidate the regulatory mechanisms of gene expression for long and the radical discovery of regulatory function of endogenous small noncoding RNAs is overwhelming the scientific community with their ever increasing potentials [1]. Small noncoding RNAs of 18–40 nucleotides (nt) in size have been proved to play a vital role in a remarkably wide range of biological processes, including cell proliferation, developmental timing and patterning, chromatin modification, genome rearrangement, and stress response in plants and animals [2]. Small RNAs regulate a variety of biological processes in plants by interfering with messenger RNA (mRNA) translation, directing mRNA cleavage or promoting the formation of compact, transcriptionally inactive chromatin [3]. Several distinct classes of small RNAs have been reported so far including microRNAs (miRNAs) [4], small interfering RNAs (siRNAs) [5], repeat-associated small interfering RNAs (ra-siRNAs) [6], Piwi interacting RNAs (piRNAs) [7], natural antisense transcript derived small interfering RNAs (nat-siRNAs) [8], transacting small interfering RNAs (ta-siRNAs) [9], heterochromatic small interfering RNAs (hc-siRNAs) [10], secondary transitive small interfering RNAs, primary small interfering RNAs, competing endogenous RNAs (ceRNAs), and long small interfering RNAs [11–13]. It has only been a few years since it was appreciated that microRNAs provide an unanticipated level of gene regulation in both plants and metazoans [11].

miRNAs are well differentiated due to some of their particular characteristics [12]; they are derived from distinct genomic loci and processed from transcripts that can form local RNA hairpin structures, and usually miRNA sequences are nearly always conserved in related organisms [13, 14]. Most miRNAs are transcribed by RNA polymerase II which folds into a stable, usually imperfect, hairpin structure [13]; pri-miRNA transcript is cleaved to pre-miRNA by RNaseIII-type Dicer-like 1 (DCL1) protein [15] to produce a distinctive ~21 nt, double-stranded RNA. This duplex is exported into the cytoplasm by HASTY and methylated at the 3′ end by HEN1 [16]. A cytoplasmic helicase unwinds the translocated duplex into a single-stranded mature miRNA, which is finally incorporated into RNA-induced silencing complex (RISC) [5, 17, 18]. A mature miRNA sequence can range from 19 to 24 nucleotides (nt) in length and act as a regulatory molecule in posttranscriptional gene silencing by base pairing with target mRNAs [12]. Within the RISC complex, miRNAs function in the direct cleavage of 3′ untranslated region of protein-coding genes or translational repression depending on its perfect or imperfect match with the targets [19]. The same mature miRNA can also be present as several length variants; these populations of miRNA variants are called isomiRNAs, which are isoforms of microRNAs caused by an imprecise or alternative cleavage of Dicer during pre-miRNA processing [20].

Several miRNAs have been identified in plants, and they have been characterized in a wide variety of metabolic and biological processes with important functions [12]. The first plant miRNAs were described in* Arabidopsis thaliana* [21]; currently the latest miRBase release (v20, June 2013) contains 24,521 microRNA loci from 206 species, processed to produce 30,424 mature microRNA products [22]. Earlier, miRNAs have been identified through either bioinformatics analysis or sequencing [23]; various methods have been used to identify miRNAs in rice [24], wheat [25], tomato [26], and maize [27]. Besides the miRNAs that are highly conserved in different species, there are species-specific miRNAs originating from recently evolved miRNA genes [28, 29]. The expression of these species-specific miRNAs is often low and can therefore be difficult to detect by traditional methods [30]. In recent times, high-throughput sequencing platforms are showing significant promise for small RNA discovery and genome-wide transcriptome analysis at single-base pair resolution [23, 31]. Sequencing techniques such as the Solexa Platform, SOLiD, and 454 Technology as well as other massively parallel sequencing strategies have been successfully applied in order to identify miRNAs in many plant species, such as rice [32], alfalfa [33], grape [34], tomato [35], orange [36], soybean [37], peanut [38], poplar [39], and black gram [40]. In comparison with microarray, deep sequencing has several advantages, the major one being its application in comprehensive identification and profiling of small previously unknown RNA populations [23]. Nevertheless, analyses of these data are not perfect, especially in the absence of native genome sequence [40].

Jute (*Corchorus olitorius* and* Corchorus capsularis*) is a bast fibre, like flax and hemp. Cultivation of this environmentally friendly as well as the most affordable fibre producing plant is concentrated around the Ganges Delta region of Bangladesh and India where the warm, wet climate during the monsoon season provides ideal growing conditions. In terms of usage, production, and global requirement, jute is second only to cotton [41]. Jute plants are easy to grow, have a high yield per acre, and, unlike cotton, have little need for pesticides and fertilizers. They are also known to enrich the soil [42]. As these plants grow fast, they are often used in crop rotation. The leaves and roots left after harvest enrich the soil with micronutrients, maintaining soil fertility. When used as a geotextile, it puts nutrients back in the soil when it decomposes. This rain-fed crop during its growth helps to clean the air by assimilating three times more CO_2_ than an average tree, converting the CO_2_ into oxygen.

Despite its great agronomic importance, research on jute at the molecular level is insignificant [43]. Genome sequence of jute is not available in the public database. So far only 1,210 sequences are found in the GenBank [43] with no deposits of any miRNA sequences in miRBase database. Within this context, the current study has employed the deep sequencing strategy in an attempt to effectively identify conserved and novel jute miRNAs. Quantitative real-time PCR (qRT-PCR) has been performed to determine the expression of these miRNAs. For mapping the identified miRNAs, genome of* Vitis vinifera* was used as reference because of sequence homology of this species with jute [44].

## 2. Methods and Materials

### 2.1. Plant Materials Preparation and Small RNA Library Construction

Seeds of farmer popular O-9897 variety of tossa jute (*Corchorus olitorius*) were collected from Bangladesh Jute Research Institute (BJRI). They were surface sterilized with 70% ethanol, subsequently washed in distilled water, and allowed to germinate on sterile petri dishes containing 3 MM moist filter paper (Whatman) at 30 ± 1°C and 65% relative humidity. Seeds were allowed to grow for 4 days under the specified conditions. On the fourth day of germination, seedlings were collected and immediately snap-frozen in liquid nitrogen and stored at −80°C for subsequent use.

RNA was isolated from collected seedlings using TRIzol reagent (Invitrogen, USA) by following the manufacturer's instructions. Later, RNA samples were sent to Beijing Genome Institute (BGI, Shenzhen, China) for deep sequencing of small RNA by Illumina Hiseq high-throughput sequencing platform. In short, the sRNAs pool of 18–30 nt in length were fractionated from total RNA. After ligation with 5′ and 3′ adaptors, the short RNAs so obtained were reverse-transcribed to cDNA according to the Illumina protocol. The resulting small RNA library was then sequenced following SBS method (sequencing by synthesis) by Illumina Hiseq high-throughput sequencing.

### 2.2. Prediction of Known miRNA in Jute

Raw data obtained from Illumina Hiseq high-throughput sequencing was at first filtered by removing contaminants which include low quality reads, reads with 5′ primer contaminants, reads without 3′ primer, reads without the insert tag, reads with poly A, and reads shorter than 18 nt. After cleaning, the final reads were then used for further analyses. Clean reads fully matching other RNAs, including mRNA, rRNA, tRNA, snRNA, snoRNA, and repeat RNA, were excluded by using BLASTn-short alignment (blast2.2.26+, ftp://ftp.ncbi.nih.gov/blast/executables/blast+/2.2.26/) and aligning against Sanger RNA family database (Rfam 11.0, ftp://ftp.sanger.ac.uk/pub/databases/Rfam). The remaining unique sequences were further aligned against miRBase-v20 [22] allowing up to 3 mismatches to identify known miRNAs present in* C. olitorius*. Mature miRNAs present within a genome encoding identical or nearly identical sequences were then grouped together into a family.

### 2.3. Novel miRNA Identification

Prediction of novel miRNA was done using prediction software, Mireap (http://sourceforge.net/projects/mireap/), developed by BGI by taking into consideration secondary structure, cleavage position of Dicer protein, and minimum free energy of the unannotated small RNA tags. Strategic conditions for selecting unique miRNA are as follows: (i) the tags which are to be used to predict novel miRNA should be from unannotated tags which can match reference genome (*Vitis vinifera*), from the tags which align to introns as well as antisense exons; (ii) genes, whose sequences satisfy the above standards and their secondary structures which allow hairpin miRNAs to fold within them and the presence of mature miRNAs in one arm of the hairpin precursors, are considered as candidate genes for miRNA; (iii) the possible candidate mature miRNA strand contains 2-nucleotide 3′ overhang; (iv) hairpin precursors of the candidate miRNAs are devoid of large internal loops or bulges; (v) secondary structures of the hairpins are stable, with the minimum folding energy (MFE) lower than or equal to −20 kcal/mol.

### 2.4. Target Gene Prediction

The rules used for target prediction in plants are based on those suggested by Allen et al. [9] and by Schwab et al. [45]. These are (i) presence of maximum 4 mismatches between small RNA and target (G-U bases count as 0.5 mismatches), (ii) not more than 2 contiguous mismatches in the miRNA/target duplex, (iii) no end-to-end mismatches at the 5′ of miRNA from 2 to 12 positions of the miRNA/target duplex, (iv) no mismatches in positions 10-11 of miRNA/target duplex, (v) a maximum of 2.5 mismatches in positions 1–12 of the miRNA/target duplex from 5′ region of miRNA, and (vi) minimum free energy (MFE) of the miRNA/target duplex which should be ≥ 74% of the MFE of the miRNA bound to its perfect complement. The targets of miRNAs were further validated by a well-recognized miRNA-target prediction tool: psRNA Target [46]. In addition to potential target prediction for known and novel miRNAs, pathways which include the corresponding target genes as well as the biological function of such genes are taken into consideration by using the grape genome as a reference. Biological functions are recommended by using GO (level 3) (http://www.geneontology.org/). Gene Ontology (GO) is an international standard classification system for gene function, which provides a set of controlled vocabulary to comprehensively describe the property of genes and gene products [47]. There are 3 ontologies in GO: biological process, cellular component, and molecular function, containing lists of biological functions that illustrate each gene and its product (http://www.geneontology.org/). Each category defines precise participation of a given gene within an organism. As for pathway identification, KEGG (http://www.genome.jp/kegg/) [48] database was used to culminate the target genes within the systematic biological pathways. KEGG analyses reveal the main pathways with which the target gene candidates are involved [49].

### 2.5. Validation of the Presence of Jute miRNAs

To verify the identified known and potential novel miRNA candidates in jute, stem-loop reverse transcription-PCR was performed [50].

Stem-loop primers were designed according to the method described by Chen et al. [51]. This primer binds to specific miRNA at the 3′ region owing to the precision conferred by the primer with the exact reverse complement of six nucleotides from the 3′ end of each particular miRNA sequence, which is reverse-transcribed by the RT enzyme. Two thousand nanograms of total RNA were used to perform the RT reaction with Superscript III First Strand Synthesis System (Invitrogen, USA) according to the protocol of Varkonyi-Gasic et al. [50], which has been further standardized for jute. For a reaction volume of 20 *μ*L, 0.5 *μ*L of 10 mM dNTPs was at first taken together with an appropriate amount of RNA and an adjusted amount of DEPC-treated H_2_O followed by heating the mixture for five minutes at 65°C and then immediately transferring the same on ice. While keeping on ice for approximately 2 minutes, 4 *μ*L of 5X FS buffer, 2 *μ*L of 0.1 M DTT, 1.2 *μ*L of 1 *μ*M stem-loop primer, 0.25 *μ*L of M-MLV Superscript III RT (200 U/*μ*L), and 0.1 *μ*L RNaseOUT (40 U/*μ*L) were added to the reaction mix. The RT reaction was carried out in a thermal cycler (Mastercycler, Eppendorf, Germany), followed by a pulse RT cycle starting from incubation at 16°C for 30 minutes, then 60 cycles of 30 sec. at 30°, 30 sec. at 42°C, and 1 sec at 50°C. This step was followed by another incubation step of 5 mins at 85°C to inactivate the RT enzyme.

End point PCR was then conducted with miRNA specific forward primer and a universal reverse primer to check the presence of the specific miRNAs. PCR products were electrophoresed on 3% agarose gel in 1X TAE and stained with ethidium bromide before visualization under a transilluminator. We further conducted quantitative real-time PCR for confirming the expression of some selected known and novel miRNAs using a 32-well plate Roche LightCycler Nano System and the Roche SYBR Green Master I (Roche Diagnostics, Germany). Briefly, equal amount of cDNA was taken in a reaction volume of 7.5 *μ*L in triplicate with 0.1875 *μ*L of each primer (forward and universal reverse) and 3.75 *μ*L of SYBR Green Master I. Thermo cycling conditions were set at an initial polymerase activation step for 600 seconds at 95°C, followed by 45 cycles of 5 sec at 95°C for template denaturation, 10 sec at 60°C for annealing, and 1 sec at 72°C for extension and fluorescence measurement. Later, a dissociation protocol with a gradient from 50°C to 95°C was used for each primer pair to verify the specificity of the RT-qPCR reaction and the absence of primer dimers.

## 3. Results

### 3.1. Deep Sequencing of Jute Small RNAs

In order to identify microRNAs in jute, RNA was isolated from the total tissue of four-day seedlings and subjected to Illumina Hiseq high-throughput sequencing by synthesis (SBS) technology. Among a total of 16912862 raw reads, 16822412 high quality reads were filtered through a series of data cleaning processes. The details of tag cleaning are summarized in Table 1, which shows that a total of 16644324 clean reads were obtained by removing 3′ adapter null, insert null, 5′ adapter contaminants, sequences smaller than 18 nt, and poly A.

**Figure 1:**
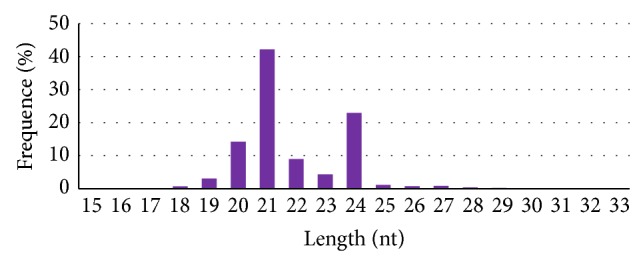
Length distribution of small RNAs found in jute (violet bar indicates the percentage of total tags).

This comprises about 99% of the high quality reads. Distribution of these clean reads that contain a pool of small RNAs ranging from 18 to 30 nucleotides is shown in Figure 1. However, the sizes of small RNAs were not found to be uniform; majority (92.69%) of the sRNAs are 20–24 nt in size, with 21 nt being the most abundant (42.19%), followed by 24 nt (22.95%) and 20 nt (14.25%), respectively. These sequences were then aligned to Rfam 11.0 [52] and Genbank database (BLASTn) to identify common sRNAs other than miRNAs, as well as to remove mRNAs (see supplementary file-1 in Supplementary Material available online at http://dx.doi.org/10.1155/2015/125048). The remaining sequences were matched against miRBase-20 database [53] for the prediction of miRNAs, revealing 33433 unique and 8994892 redundant reads, which were finally used to identify known miRNAs. The novel miRNAs in jute were identified from unannotated tags by using Mireap, software developed by BGI (described in Section 2).

### 3.2. Identification of Known miRNAs in Jute

In the absence of the complete genome sequence and with practically no information on miRNA of jute in miRBase, clean reads were aligned to the miRNA precursor/mature miRNA of all plants in miRBase allowing up to three mismatches or free gaps [54] to identify known miRNAs. The expression of miRNA is generated by summing the count of tags which can align to the temporary miRNA database, generated by choosing the most expressive miRNA of each mature miRNA family.

**Figure 2:**
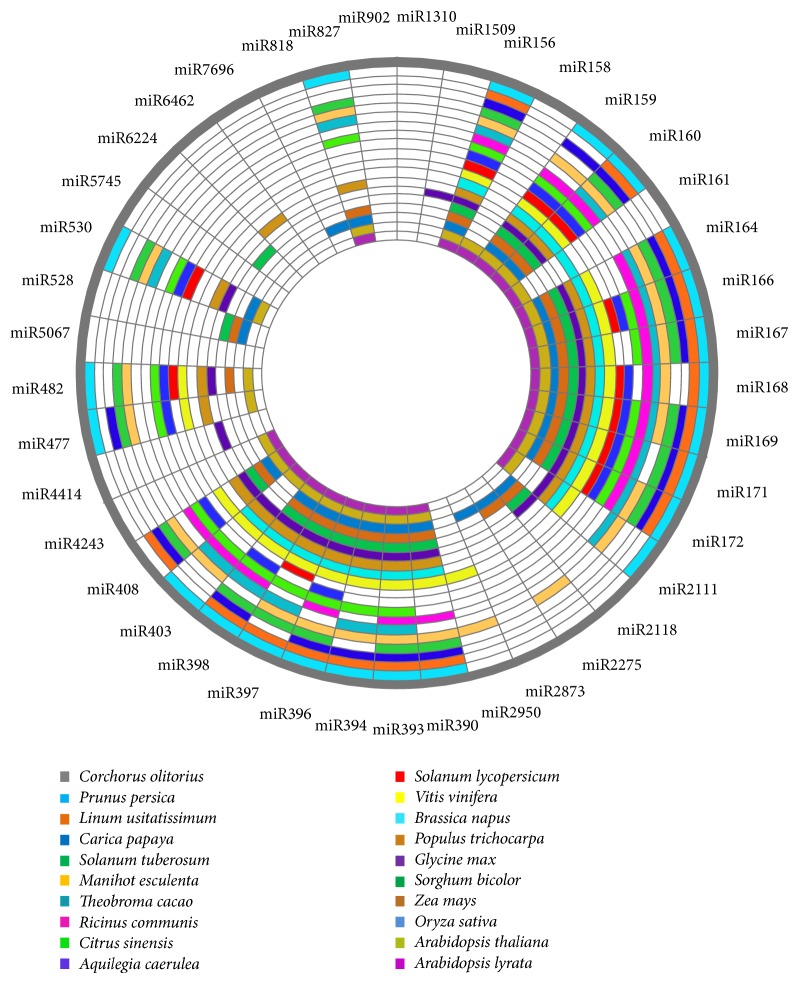
Conservancy of miRNAs identified in jute is presented as circular heat map among different model plants. Each color represents a different plant species and white color represents absence of miRNA. miRNA that was found in at least 9 plants was considered as conserved.

**Figure 3:**
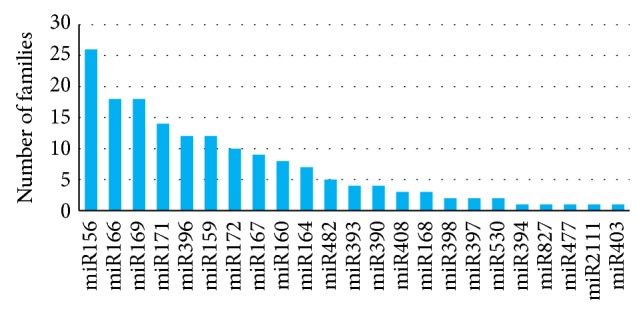
Number of family members of conserved miRNAs are represented as bar diagram.

A total of 227 known miRNAs were identified in this study, of which 164 belong to 23 conserved and 63 to 58 nonconserved families. These nonconserved families were further categorized into 18 defined and 40 undefined families. Conservancy of miRNA families found in jute showed high homology with their respective homologs in other model plants (Figure 2). However, the number of family members of conserved miRNAs was highly variable, with miR156 being the largest family, consisting of 26 members, whereas miR403, miR394, miR827, miR477, and miR2111 were the smallest among the families, comprising only one member. miR166 and miR169 were the second largest with each having 18 members and miR171 was the third largest family with 14 members (Figure 3). Most of the conserved families contain both 5p and 3p mature miRNA sequences, attaching a high confidence to the data set [54]. Highly variable reads numbers were also found among the families, even in members of the same family, indicating different expression levels of these miRNAs. Among them, col-miR157a had the highest level of expression, having 5531609 counts, and the other miRNAs like col-miR156a, col-miR166a, and col-miR167h also had relatively high reads numbers, counting more than 150000. Several conserved miRNAs (like miR171, miR398, and miR159) and, as expected, most of the nonconserved miRNAs had relatively low copy numbers. Interestingly, a miRNA named col-miR3954 from an undefined family had very high level of expression, having 868222 reads, third highest of all miRNAs found in jute. miRNAs from each family with highest reads number are shown in Table 2. High expression frequency of miRNAs derived from the 3′ arm of some pre-miRNAs, like col-miR166h-3p, col-miR166g-3p, col-miR166j-3p, col-miR165a-3p, col-miR396b-3p, col-miR396e-3p, and so forth, compared to their corresponding 5′ arm-miRNAs, supports the observations of functional activity of both arms of pre-miRNA hairpins [55, 56]. Details of the miRNAs found in jute are summarized in supplementary file-2.

### 3.3. Identification of Novel miRNAs

The miRNA hairpins are mostly located in intergenic regions, introns, or reverse repeat sequence of coding sequences [57]. Thus tags belonging to these regions were used to predict novel miRNAs. Characteristic hairpin structure of miRNA precursor was used to predict novel miRNA with prediction software Mireap (http://sourceforge.net/projects/mireap/) by exploring the secondary structure, the Dicer cleavage site, and the minimum free energy of the unannotated small RNA tags which could be mapped to the reference* Vitis vinifera* genome. Predicted secondary structures were further validated by Mfold (supplementary file-3) [58] and novel miRNAs were identified based on the selection criteria described in Section 2. 17 potential novel miRNAs have been identified in this study of which 9 miRNAs were derived from 3′ arm of the pre-miRNA sequences and 8 from the 5′ arm (Table 3). Average length of the pre-miRNAs sequences ranged from 78 to 349 nt, similar to those found in maize [59] and rice [24]; minimum folding energy (MFE) for jute miRNAs was observed to be within a range from −21 to −105.3 kcal/mol, similar to the range observed in cucumber [60] (supplementary file-4). Expression of novel miRNA was determined by summing the count of such miRNAs which have no more than 3 mismatches on either the 5′ or 3′ ends and with no mismatch in the middle. Novel miRNAs usually have lower levels of expression than the conserved miRNAs, as evident from findings of several plant species like soybean,* Brassica napus*, maize,* Arabidopsis*, and wheat [59, 61–64].

### 3.4. Target Gene Prediction for Identified miRNAs

**Figure 4:**
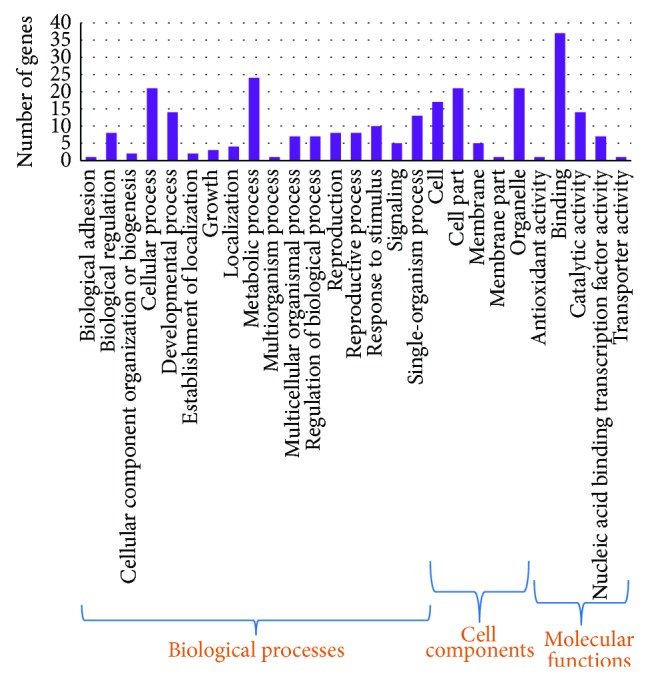
GO (level 3) annotation of predicted targets. Violet bar indicates the number of targets involved in each process.

For a precise elucidation of the role of miRNAs, target identification and determination of their biological functions are of vital importance. With a plethora of experimentation, it is now evident that cleavage or translational repression site of most known plant miRNAs is located in the CDS (coding sequence) region of their target mRNA with perfect or nearly perfect sequence complementarity [65], making it feasible to identify plant miRNA targets [4, 21, 66]. In this study, target genes of miRNAs were identified by BLASTn against the genome sequence of* Vitis vinifera*, following methods described by Allen et al. and Schwab et al. [9, 45]. Among a total of 79 identified miRNA (both conserved and nonconserved) families, 116 potential target genes were predicted for 39 families (supplementary file-5). A total of 46 genes from this prediction overlapped with targets identified by psRNA Target which found 99 target genes for 19 miRNA families (supplementary file-9). Highest number (16) of targets was identified for miR397 family, all of which are laccase, an enzyme, involved in plant cell wall lignification [67]. miR3946 had the second highest number of targets with 11 genes. Most of the other families targeted only a single gene. For the novel jute miRNAs, a total of 11 targets were predicted for 4 among the 17 identified miRNAs (Table 4, details in supplementary file-6), with a maximum number of target genes (6) recognized for col-miRN7. Most of col-miRN7 targets are NB-ARC domain containing protein, which is a resistance (*R*) protein, involved in pathogen recognition and subsequent activation of innate immune responses [68]. To better understand the functions of miRNAs, target genes were analyzed by Gene Ontology (GO) level 3 to divulge the regulatory network of miRNAs and target genes [69]. Such analysis demonstrates that for jute 133 predicted target genes (both for known and novel miRNAs) can be classified into 20 having biological, 5 cellular, and 5 molecular functions. Same gene was found to be involved in multiple processes with the reverse being also true (Figure 4 and supplementary file-7). As illustrated by KEGG pathway analysis (supplementary file-8) [70], the predicted target genes of jute miRNAs were found to be involved in 42 different pathways.

### 3.5. Validation of the Presence of Known and Novel miRNAs in Jute

**Figure 5:**
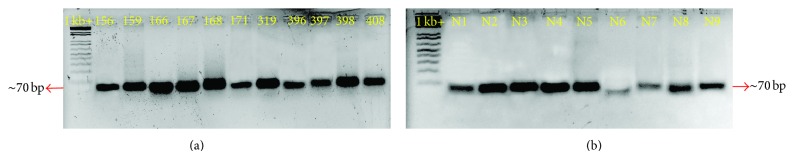
Agarose gel electrophoresis of known and novel miRNAs identified in jute. (a) Amplicons for known miRNAs. 156: miR156, 159: miR159, 166: miR166, 167: miR167, 168: miR168, 171: miR171, 319: miR319, 396: miR396, 397: miR397, 398: miR398, and 408: miR408. (b) Amplicons for novel miRNAs. N1: col-miRN1, N2: colmiRN2, N3: colmiRN3, N4: colmiRN4, N5: colmiRN5, N6: colmiRN6, N7: colmiRN7, N8: colmiRN8, and N9: colmiRN9.

**Figure 6:**
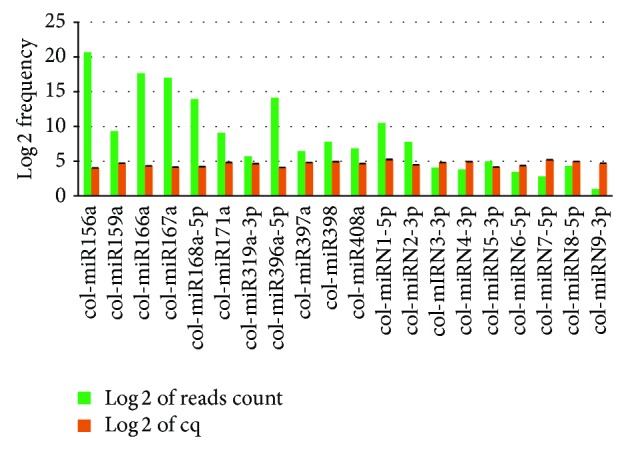
Comparative expression analysis of different selected miRNAs found by deep sequencing and qRT-PCR. Read counts of deep sequencing and cq values of qRT-PCR were converted into log 2 value for a better representation. Here the green bars represent log 2 values of sequencing frequency and orange bars represent the log 2 values of cq produced by qRT-PCR. Black regions on top of the orange bars represent errors calculated as standard deviation.

Some of the miRNAs identified through deep sequencing were verified by the standard stem-loop RT-PCR method [50] followed by end point PCR and qRT-PCR. The stem-loop primers were designed with a 3′ specificity for a particular miRNA which hybridizes to the same and is reverse-transcribed by the RT enzyme. These primers increase the sensitivity of the reactions such that this method can significantly distinguish two miRNAs with only one single nucleotide change [51]. The RT product is then subjected to end point and qRT-PCR. Forward primers were precisely designed from the first 15 bases of each miRNA with 5′ extension of random GC rich sequence to increase the melting temperature as mentioned by Varkonyi-Gasic et al. in 2007 while the reverse primer is a universal sequence that is designed from the 5′ region of the stem-loop RT primer [71]. A set of 11 randomly selected conserved miRNAs as well as 9 novel miRNAs were used for verification. In this study, the stem-loop primer used was 50 bp long together with 5′ (forward primer) and 3′ extensions, and the end point PCR product size ranged from 60 to 70 bp. Amplification of the product gave a sharp band for each of the selected known and novel miRNAs (shown in Figure 5). cDNAs were further amplified by qRT-PCR in technical triplicates from which log 2 values of Cq were calculated for each of the miRNAs and average of these values was compared with the log 2 value of read counts obtained from deep sequencing. Most of the qRT-PCR results acceded with the sequencing data; however, in some cases, discrepancy was observed (Figure 6).

## 4. Discussion

Widespread discovery of miRNAs and their critical role in gene regulation has made it ever important to recognize them in different species. Identification of miRNAs and their targets is the basis for understanding their physiological functions [60].

While a large amount of miRNAs are reported and deposited in databases from different plants, miRNA associated research in jute is still to be instigated. Without the genome sequence of jute at hand, identification of miRNA and their targets in jute by deep sequencing of small RNAs has been the greatest challenge of the current study. Use of closely related species' genomes as proxy references can facilitate miRNA identification in nonmodel species like jute for which genome sequence is not available [72]. We have used the grape genome as the background because of sequence similarity between these two species.

sRNAs with known function are commonly 20–24 nt in size [34]. Analyses of size distribution patterns of the reads show that the most abundant sRNAs in jute are 21 nt in size which is about 42.19%, consistent with recent identification of sRNAs in different plant [34, 62, 64].

Sequencing frequencies for miRNAs in a library can be used as an index for estimating the relative abundance of miRNAs [73]. Numerous small RNA sequences, engendered from Illumina Hiseq high-throughput sequencing platform, show the presence of different miRNA families and are even able to differentiate between distinct members of a given family. miR156 family which is highly conserved across the species [74] was found to be the largest family in jute seedlings with the highest expression of col-miR157a followed by col-miR156a. Two other members of the same family, namely, col-miR156c and col-miR156k, also show significant levels of expression. During shoot development, miR156 regulates the transition of plants from juvenile to adult phase by targeting SPL genes [75]. In* Arabidopsis*, miR156 is strongly expressed during seedling development and shows weak expression in mature tissues [76]. This could explain the relative abundance of the members of miR156 family since RNA used in sequencing was extracted from jute seedlings. Deep sequencing technology allows distinguishing and measuring miRNA sequences with only a few nucleotide changes [38]. Members of different families exhibit considerably dissimilar expression levels. For example, the abundance of miR156 family varied from 1 read (col-miR156p) to 5531609 reads (col-miR157a). This was also the case for some other miRNA families, such as col-miR166 (from 3 to 215636 reads) and col-miR167 (from 12 to 154973 reads). Presence of a prevailing member in a miRNA family may indicate the dominant role of this member during the growth phase at which the samples were collected. It is also to be noted that most of the conserved miRNA families consist of more than one member, whereas nonconserved miRNAs identified in this study are mostly represented by a single MIR (miRNA) gene.

It has been hypothesized that MIR genes originate by gene duplication events followed by random mutation processes to evolve in multiples of imperfectly paired hairpins [77, 78]. Consequently, ancient evolutionarily conserved miRNAs are represented by multiple MIR genes whereas nonconserved miRNAs (believed to be evolutionarily recent) generally originate from a single locus [79]. It is plausible that the conserved miRNAs are responsible for control of basic cellular and developmental pathways common to most eukaryotes whereas nonconserved miRNAs are involved in regulation of species-specific pathways and functions [80].

Species-specific miRNAs are believed to have recently evolved and, in general, expressed at levels lower than those of strictly conserved miRNAs [34, 77]. Data acquired from sequencing frequencies of conserved and nonconserved miRNAs fits well with this extrapolation, where the nonconserved and species-specific miRNAs show residual accumulation in the tested tissue. However, one miR-3954, a single member of an undefined family, appears to be expressed in significantly high levels. Its only homolog deposited in miRBase v20 is in* C. sinensis* [81], showing high frequency of reads. Though not deposited in miRBase, it has been reported in* X. sorbifolia* [82].

17 new jute specific miRNAs identified in this study show a size anticipated for sRNAs derived from DCL1 processing, although sequence variants that possess shortened or extended 5′ or 3′ ends were also found. Ten among the seventeen new col-miRNAs are 21 nt in size, consistent with canonical DCL1 products [79]. However length variation was also found. Two, col-miR2 and col-miR9, are 20 nt in size; three, col-miR5, col-miR6, and col-miR14, are 22 nt long. col-miR13 was found to be 23 nt in size which can probably be explained by the fact that diverse miRNA families are also independently processed by DCL3 to generate a new class of bona fide (23–25 nt) miRNAs with no canonical size, called long miRNAs [83].

A total of 20 miRNAs of both conserved and species-specific origin were corroborated by stem-loop RT-PCR and their expression pattern was assessed by qPCR to validate the data obtained from deep sequencing. Discrepancies in the expression pattern of some miRNAs found by deep sequencing and qPCR can be attributed to practical differences between the sensitivity and specificity of these two techniques [84]. The sensitivity and large dynamic range of next generation sequencing (NGS), along with its consistent prediction of fold changes when compared with gold-standard qPCR, support its use for discovery-oriented and exploratory miRNA profiling experiments [84, 85].

To evaluate and outline a putative function for a miRNA in plants, target identification is necessary [73]. We have predicted target genes for known and potential new miRNAs identified in this study using the genome of* Vitis vinifera* as a reference. Most of the target genes for conserved miRNA families predicted in jute have already been confirmed in model plants, as target genes are commonly conserved [78, 80]. miR156/157-Squamosa promoter-binding protein [86], miR166-Homeodomain Leucine Zipper protein III (HD-ZIP III) [87], miR167-auxin response factor (ARF) [88], miR164-NAC domain protein [89], miR172-transcription factor APETALA2 [90], miR159-MYB transcription factor [91], miR171-GRAS family transcription factor [92], miR394-F-box family protein [93], and miR395-ATP sulfurylase [94] well characterized miRNA-target pairs in other plants have been found in jute. However, a number of widely studied miRNA-target pairs, such as miR398-copper superoxide dismutase [95], miR399-E2 ubiquitin conjugating protein [96], and mir162-Dicer-like 1(DCL1) [97], were not found in this study. This could possibly be due to the fact that the jute genome sequence is not available to be used as a reference. However, conserved miRNAs with their nonconserved targets, including miR167-peroxidase29, miR396-eukaryotic translation initiation factor 2c, miR168-NAC domain containing protein, miR164-growth regulating factor 1, miR390-AP domain containing transcription factor, miR160-MYB transcription factor, and miR393-GTP-binding protein alpha subunit, were also found to be present in jute, allowing presumption of nonconserved targets for conserved miRNAs. Highest number of target genes were identified for miR397, which is laccase, a well-studied enzyme, encoded by multigene families in poplar,* Arabidopsis*, rice, and* Liriodendron tulipifera* [98], reported to be involved in lignin biosynthesis of plants [99–101]. High lignin content of jute fibre limits its use in making fine fabrics [102]. Toughness of this biopolymer also poses a major obstacle to pulping, forage digestibility, and biofuel production [103]. It has been reported that transgenic* P. trichocarpa* plants overexpressing Ptr-miR397a result in a reduction of Klason lignin content [104], supporting the idea that use of miR397 would be an attractive means for reducing lignin-related problems. Future experiments including in-depth studies of miR397-laccase pair may help in producing quality products from jute.

## 5. Conclusion

This is the first report on jute miRNA identification. This set of experimentations for identification of miRNAs and their potential targets can initiate further study on understanding the mechanisms of regulation of jute miRNA.

## Supplementary Material

Supplementary file-1 contains total and unique reads of different RNAs that have been sequenced. Supplementary file-2 contains all the known miRNAs in jute seedling that have been found in this study. Supplementary file-3 covers the secondary structures of novel miRNA in jute predicted using Mfold. Supplementary file-4 holds the detail of novel jute miRNAs. Supplementary file-5 contains the detail of predicted targets for some of the known miRNAs. Supplementary file-6 covers the detail of predicted targets for novel miRNAs. Supplementary file-7 contains the biological functions of predicted targets. Supplementary file-8 contains the detail of pathways for predicted miRNAs. Supplementary file-9 contains the validation of predicted targets by another tool.

## Figures and Tables

**Table 1:** Summary of data cleaning.

Type	Count	Percent (%)
Total_reads	16912862	
High_quality	16822412	100%
3′ adapter_null	12978	0.08%
Insert_null	1934	0.01%
5′ adapter_contaminants	86857	0.52%
Smaller_than_18 nt	74711	0.44%
Poly A	1608	0.01%
Clean_reads	16644324	98.94%

**Table 2:** miRNAs from each family with the highest frequency in jute with their homologs in other plants.

Jute family	Jute miRNA	Length of small RNA sequence	Count of small RNA sequences	Small RNA sequence	Homolog (best one)	Matches number	Mismatches number	Gaps number
Conserved								
miR156	col-miR157a	21	5531609	UUGACAGAAGAUAGAGAGCAC	ath-miR157a	21	0	0
	col-miR156a	20	1724997	UGACAGAAGAGAGUGAGCAC	ath-miR156a	20	0	0
miR166	col-miR166a	21	215636	UCGGACCAGGCUUCAUUCCCC	ath-miR166a	21	0	0
miR167	col-miR167h	22	154973	UGAAGCUGCCAGCAUGAUCUUA	mdm-miR167h	22	0	0
miR396	col-miR396b-3p	21	18695	GCUCAAGAAAGCUGUGGGAGA	gma-miR396b-3p	21	0	0
miR168	col-miR168a	21	16590	UCGCUUGGUGCAGGUCGGGAA	ath-miR168a	21	0	0
miR164	col-miR164a	21	7491	UGGAGAAGCAGGGCACGUGCA	ath-miR164a	21	0	0
miR169	col-miR169b	21	2528	CAGCCAAGGAUGACUUGCCGG	ath-miR169b	21	0	0
miR390	col-miR390a	21	2952	AAGCUCAGGAGGGAUAGCGCC	ath-miR390a	21	0	0
mir160	col-miR160a-3p	21	1448	GCGUAUGAGGAGCCAAGCAUA	gma-miR160a-3p	21	0	0
miR159	col-miR159a	21	484	UUUGGAUUGAAGGGAGCUCUA	ath-miR159a	21	0	0
miR171	col-miR171b	21	558	UGAUUGAGCCGUGCCAAUAUC	osa-miR171b	21	0	0
miR403	col-miR403	21	282	UUAGAUUCACGCACAAACUCG	ath-miR403	21	0	0
miR398	col-miR398	21	262	GGAGCGACAUGAGAUCACAUG	hbr-miR398	20	1	0
miR482	col-miR482b	22	2206	UCUUACCUACUCCACCCAUGCC	ghr-miR482b	21	1	0
miR408	col-miR408	21	129	AUGCACUGCCUCUUCCCUGGC	ath-miR408	21	0	0
miR397	col-miR397a	21	106	UCAUUGAGUGCAGCGUUGAUG	ath-miR397a	21	0	0
miIR530	col-miR530a	21	81	UGCAUUUGCACCUGCACCUUU	csi-miR530a	20	1	0
miR393	col-miR393b-3p	21	61	AUCAUGCGAUCCCUUCGGAAU	stu-miR393-3p	20	1	0
miR394	col-miR394a	20	16	UUGGCAUUCUGUCCACCUCC	ath-miR394a	20	0	0
miR827	col-miR827a	21	22	UUAGAUGACCAUCAACAAACA	ghr-miR827a	21	0	0
miR477	col-miR477i	21	2	ACUCUCCCUCAAGGGCUUCCG	mes-miR477i	21	0	0
miR2111	col-miR2111a	21	15	UAAUCUGCAUCCUGAGGUUUG	ptc-miR2111a	21	0	0
miR172	col-miR172a	21	1480	AGAAUCUUGAUGAUGCUGCAU	ath-miR172a	21	0	0

Nonconserved								
miR2275	col-miR2275a-3p	22	54	UUAAGUUUUCUCCAAUAUCUCA	zma-miR2275a-3p	20	1	2
miR2118	col-miR2118a-3p	22	36	UUGCCGAAUCCGCCCAUUCCGU	gma-miR2118a-3p	19	2	1
miR528	col-miR528-5p	21	17	UGGAAGGGGCAUGCAGAGGAG	osa-miR528-5p	21	0	0
miR1310	col-miR1310	21	69	AGGCAUCGGGGGCGCAACGCC	han-miR1310	21	0	1
miR7696	col-miR7696a-3p	21	6	UCUGAAUCAUGAGAACUUGAG	mtr-miR7696a-3p	19	1	2
miR6224	col-miR6224a-3p	21	4	CUGAUAAUAUAGGACGGAGGG	sbi-miR6224a-3p	19	1	2
miR6462	col-miR6462c-5p	21	28	AAGGGACAAAAAGGCUAUAAG	ptc-miR6462c-5p	20	0	3
miR4243	col-miR4243	21	3	UUGAACUUGUACGAUUUCGAC	ath-miR4243	19	1	2
miR5745	col-miR5745b	21	16	UUUAAUUUAUAUACAUCUCAC	mtr-miR5745b	20	0	2
miR902	col-miR902j-5p	24	1	AUAUGUUACGCAGAUUCUUCAUUU	ppt-miR902j-5p	21	0	3
miR2873	col-miR2873b	21	10	UUGUGGCUGAGAUUUGGUAUG	osa-miR2873b	19	1	2
miR2950	col-miR2950	21	2465	UGGUGUGCAGGGGGUGGAAUA	ghr-miR2950	21	0	0
miR5067	col-miR5049c	24	3837	GGACAAUUAUUGUGGGACGGAGGG	hvu-miR5049c	21	2	1
miR818	col-miR1436	23	920	AGAUAAUAUGGGACGGAGGGAGU	osa-miR1436	20	1	2
miR4414	col-miR4414a-3p	21	11	AUCCAACGAUGCAGGAGCUGC	mtr-miR4414a-3p	20	1	0
miR1509	col-miR7122a	22	488	UUGGACAGAGAAAUCACGGUCG	mdm-miR7122a	20	2	0
miR158	col-miR158a	20	34	UCCCAAAUGUAGACAAAGCA	ath-miR158a	20	0	0
miR161	col-miR161.2	21	2	UCAAUGCAUUGAAAGUGACUA	ath-miR161.2	21	0	0
Undefined	col-miR5162	24	4	AAAAUGACCAAAAUACCCCUAAAU	osa-miR5162	22	1	2
Undefined	col-miR6248	20	1	UAAUUGAGGAUGGAGGGAGU	osa-miR6248	18	2	1
Undefined	col-miR7767-3p	22	1	UAGGAUCAGGCAGCUUGAAGGU	bdi-miR7767-3p	19	2	1
Undefined	col-miR5997	21	1	UGAAACUCAAGUAGCUAAAAG	ath-miR5997	20	0	2
Undefined	col-miR5057	23	1	AAACUUUCAGAUGCAUUUUGACA	bdi-miR5057	20	1	2
Undefined	col-miR6172	21	1	UGAGACCUGUUUAAGUUAGAA	hbr-miR6172	19	1	2
Undefined	col-miR6279	20	3	UAACAAGAAUUCCAGACACA	ppe-miR6279	18	2	1
Undefined	col-miR6220-3p	23	1	AGACUUAUAAUUUGGGACGGAGA	sbi-miR6220-3p	21	2	1
Undefined	col-miR6443	21	1	UGUAUGAUCAUGAUGCUGGAG	ptc-miR6443	19	1	2
Undefined	col-miR156h	20	17	UGACAGAAGAGAGAGAGCAU	vvi-miR156h	20	0	0
Undefined	col-miR3954	22	868222	UUGGACAGAGUAAUCACGGUCG	csi-miR3954	19	2	1
Undefined	col-miR167i	20	1	UCAUGCUGGCAGCUUCACUU	gma-miR167i	20	0	3
Undefined	col-miR6300	19	4400	GUCGUUGUAGUAUAGUGGU	gma-miR6300	18	0	1
Undefined	col-miR169p	21	6	UAGCCAAGGACAACUUGCCGG	osa-miR169p	21	0	1
Undefined	col-miR894	20	5406	GUUUCACGUCGGGUUCACCA	ppt-miR894	19	0	2
Undefined	col-miR472a	22	218	UUUUCCCUACUCCUCCCAUCCC	ptc-miR472a	21	1	0
Undefined	col-miR5059	21	212	CGGUCCUGGGCAGCAACACCA	bdi-miR5059	19	1	1
Undefined	col-miR2916	22	312	GGGGGCUCGAAGACGAUCAGAU	peu-miR2916	20	2	1
Undefined	col-miR5072	21	90	CGUUCCCCAGCGGAGUCGCCA	osa-miR5072	21	0	1
Undefined	col-miR477h	22	4	ACUCUCCCUCAAGGGCUUCCAG	mes-miR477h	21	0	1
Undefined	col-miR6478	21	164	CCGACCUUAGCUCAGUUGGUA	ptc-miR6478	20	1	0
Undefined	col-miR5205b	24	306	CUUAUAAUUAGGGACAGAGGGAGU	mtr-miR5205b	23	1	0
Undefined	col-miR7505	21	31	UUCAGAAACCAUCCCCUCCUU	ghr-miR7505	20	1	0
Undefined	col-miR5077	20	378	GAUUCACGUCGGGUUCACCA	osa-miR5077	18	1	1
Undefined	col-miR5054	20	904	GUUCCCCACAGUCGGCGCCA	bdi-miR5054	17	1	2
Undefined	col-miR845c	24	28	AGGCUCUGAUACCAAUUGACGUAG	vvi-miR845c	21	0	3
Undefined	col-miR916	22	28	CGAAGGUCGUCGGUUCGAAUCC	cre-miR916	19	2	1
Undefined	col-miR161-5p.1	21	11	UUGAAAGUGACUACAUCGGGG	aly-miR161-5p.1	21	0	0
Undefined	col-miR1863	24	56	AGCUCUGAUACCAUGUUAAGCAUC	pab-miR1863	21	1	2
Undefined	col-miR7490	24	41	AGUCUGAUAAACUCCACUGACGGU	ghr-miR7490	22	1	2
Undefined	col-miR3946	21	13	UUGAGAGAAGAGAGAGAGCAC	csi-miR3946	21	0	3
Undefined	col-miR7728-5p	19	5	UUCGGAUUGAGUGGAUUUU	bdi-miR7728-5p	18	1	2
Undefined	col-miR1862f	21	1	AAGGGGUUGGUUUACUUUUGG	osa-miR1862f	18	2	1
Undefined	col-miR6171	21	11	ACUAUGGAUUGCUGAAGGUUU	hbr-miR6171	19	1	2
Undefined	col-miR5021	21	31	UAAGAAGAAUAAGAAGAAUAA	ath-miR5021	18	2	1
Undefined	col-miR5244	21	6	UAUCUGAUGAUGAUUGUUGGU	mtr-miR5244	19	2	0
Undefined	col-miR5049-3p	23	30	AAGUAAUAUGGAACGGAGGGAGU	bdi-miR5049-3p	21	2	1
Undefined	col-miR5629	23	41	UUAGGGUAGUUAACGGGUAGUUA	ath-miR5629	21	1	1

**Table 3:** Potential novel miRNAs found in jute.

miRNA name	Number of reads	Mature miRNA sequence	Mature miRNA length	MFE (kcal/mol)
col-miRN1-5p	**1448**	GUGGGCGUGCCGGAGUGGUUA	21	−28.9
col-miRN2-3p	**219**	AGAGGGACUAUGGCCGCUUA	20	−53.5
col-miRN3-3p	**17**	UCGGUUUUGAAUUAGAGACGU	21	−85
col-miRN4-3p	**14**	UGAUGAUUGUGAAGAAGAUGA	21	−66.34
col-miRN5-3p	**32**	AGAGGCUCGGUGAAAUAGACAU	22	−24.62
col-miRN6-5p	**11**	UUCGUCCCCGGCAACGGCGCCA	22	−66.6
col-miRN7-5p	**7**	UUUUUUAAUUUUUUAUUUAUC	21	−21
col-miRN8-5p	**20**	GUUGAUCAAGUUGUGGAUGGC	21	−79.32
col-miRN9-3p	**2**	AAACUUCGAAUUGGGAGGGC	20	−89.3
col-miRN10-3p	**3**	UGAAUGAUUUCGGACCAGGCU	21	−48.3
col-miRN11-3p	**2**	GUAAGAAGGGGUAGAGAAAAU	21	−34.9
col-miRN12-3p	**5**	AAGAUAGAGAGCACAGAUGAU	21	−51.1
col-miRN13-5p	**3**	GGCGCUGCCUACUCACUCGGACA	23	−40.77
col-miRN14-3p	**7**	GUGAGGCUGGUUUCACAGAGCA	22	−39.1
col-miRN15-5p	**6**	GAGUGCAGCCAAGGAUGACUU	21	−64.9
col-miRN16-5p	**4**	UCAAGGUGGAGAUUGUUAGGA	21	93.4
col-miRN17-5p	**6**	UUAUACGAUGUGGGAUAUUAC	21	−105.3

**Table 4:** Target genes for jute specific miRNAs.

miRNA name	Targets number	Target accession	Annotation	Location	Free energy
col-miRN1	1	GSVIVT01015521001	Pentatricopeptide repeat-containing protein, mitochondrial	2244, 2264	−47.30 [100.00%]
col-miRN4	2	GSVIVT01020089001	Thioredoxin H	16, 36	−25.50 [77.74%]
		GSVIVT01021522001	Protease degS	67, 87	−24.50 [74.47%]
col-miRN7	6	GSVIVT01000651001	Conserved gene of unknown function	178, 198	−13.30 [76.00%]
		GSVIVT01000655001	NB-ARC domain containing protein	1291, 1311	−13.30 [76.00%]
		GSVIVT01000657001	NB-ARC domain containing protein	1246, 1266	−13.20 [75.86%]
		GSVIVT01021549001	Conserved gene of unknown function	702, 722	−12.90 [75.00%]
		GSVIVT01035288001	Casein kinase	1464, 1484	−14.20 [81.14%]
		GSVIVT01000656001	NB-ARC domain containing protein	1300, 1320	−13.30 [76.00%]
col-miRN8	2	GSVIVT01033994001	26S proteasome regulatory particle non-ATPase subunit 8	835, 855	−30.70 [78.52%]
		GSVIVT01037657001	Aconitase	30, 043, 024	−29.30 [77.11%]
