# Supplementary material for: High-Throughput Sequencing Reveals Diverse Sets of Conserved, Nonconserved, and Species-Specific miRNAs in Jute
Source: Int J Genomics. 2015 Mar 16;2015:125048. doi: 10.1155/2015/125048 (PMC4378336; doi:10.1155/2015/125048)
Supplement: Supplementary file 1 — Supplementary file-1 contains total and unique reads of different RNAs that have been sequenced. Supplementary file-2 contains all the known miRNAs in jute seedling that have been found in this study. Supplementary file-3 covers the secondary structures of novel miRNA in jute predicted using Mfold. Supplementary file-4 holds the detail of novel jute miRNAs. Supplementary file-5 contains the detail of predicted targets for some of the known miRNAs. Supplementary file-6 covers the detail of predicted targets for novel miRNAs. Supplementary file-7 contains the biological functions of predicted targets. Supplementary file-8 contains the detail of pathways for predicted miRNAs. Supplementary file-9 contains the validation of predicted targets by another tool. [file 125048.f1.zip › Supplementary file-1.pdf]

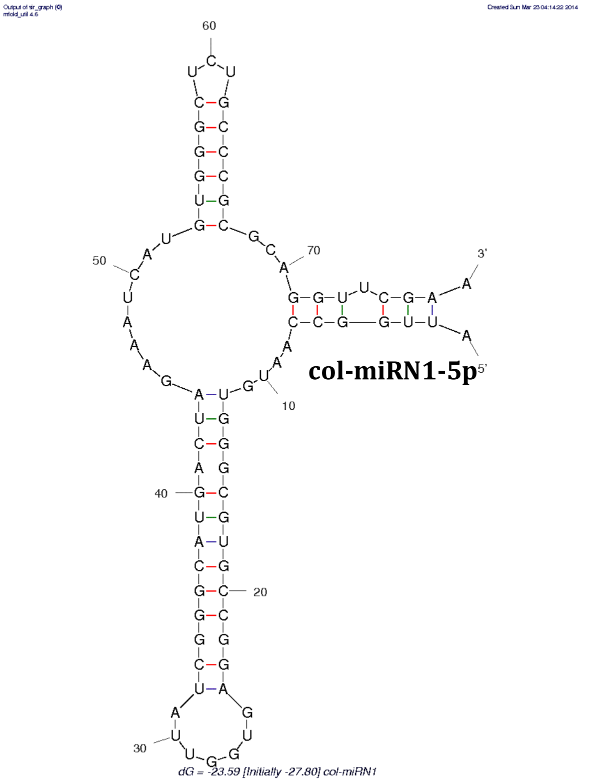
Output of sir_graph (©)
mfold_util 4.6
Created Sun Mar 23 04:14:22 2014
col-miRN1-5p
3'
5'
dG = -23.59 [Initially -27.80] col-miRN1

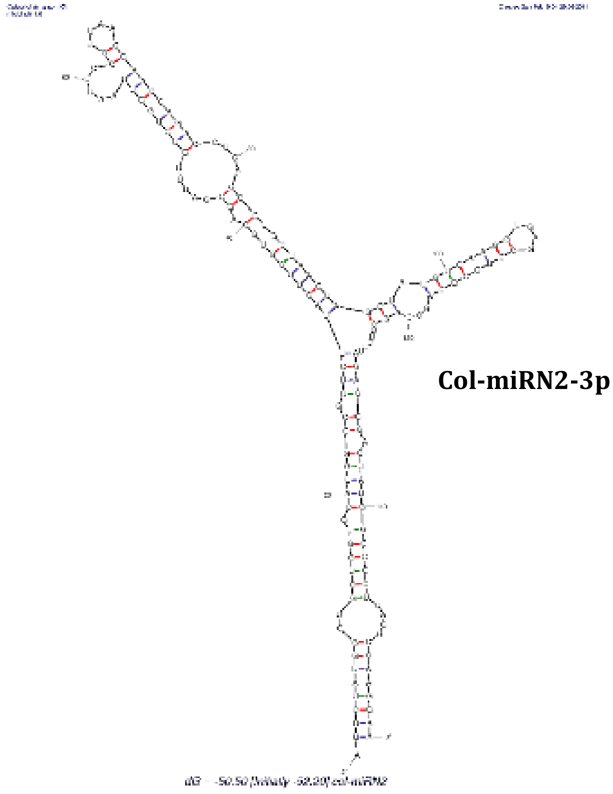
Col-miRN2-3p

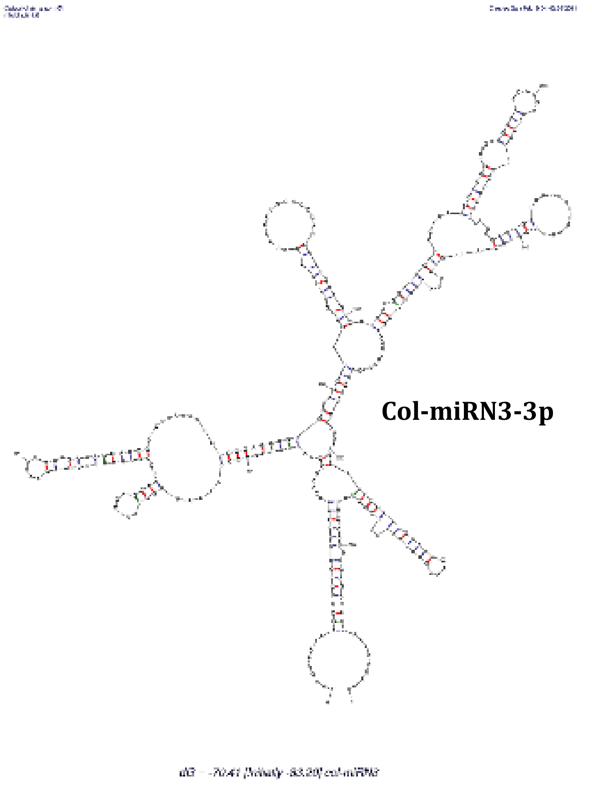
Col-miRN3-3p

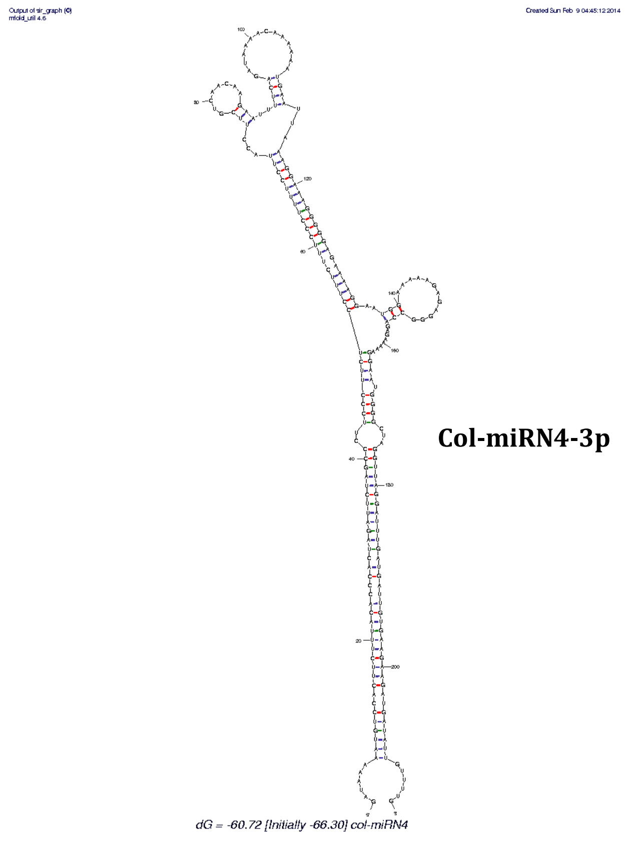
Output of sir_graph (©)
mfold_util 4.6
Created Sun Feb 9 04:45:12 2014
Col-miRN4-3p
dG = -60.72 [Initially -66.30] col-miRN4

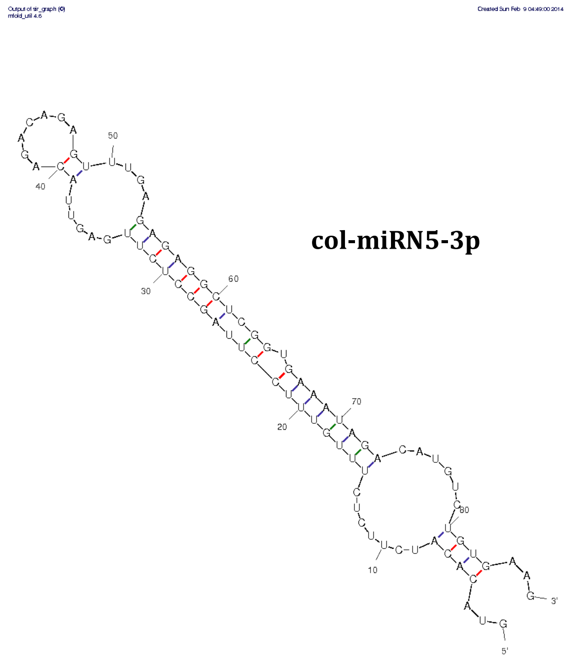

dG = -23.70 [Initially -23.70] col-miRN5

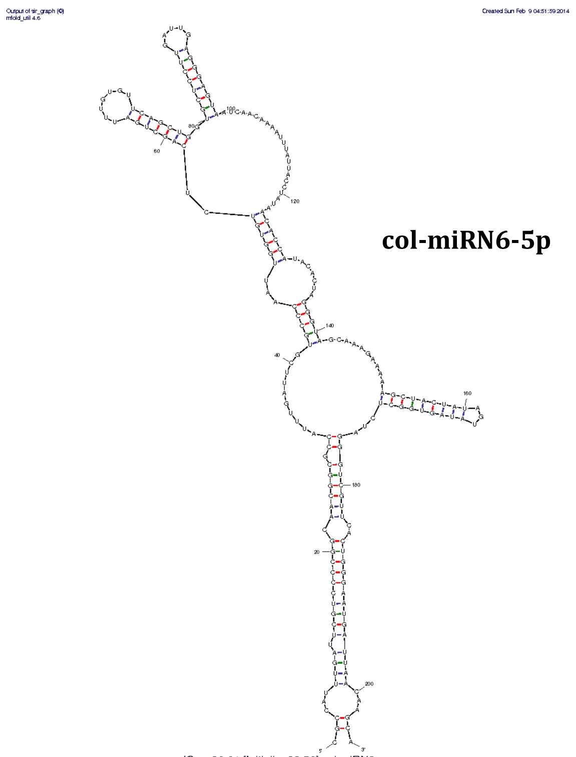

dG = -60.01 [Initially -66.50] col-miRN6

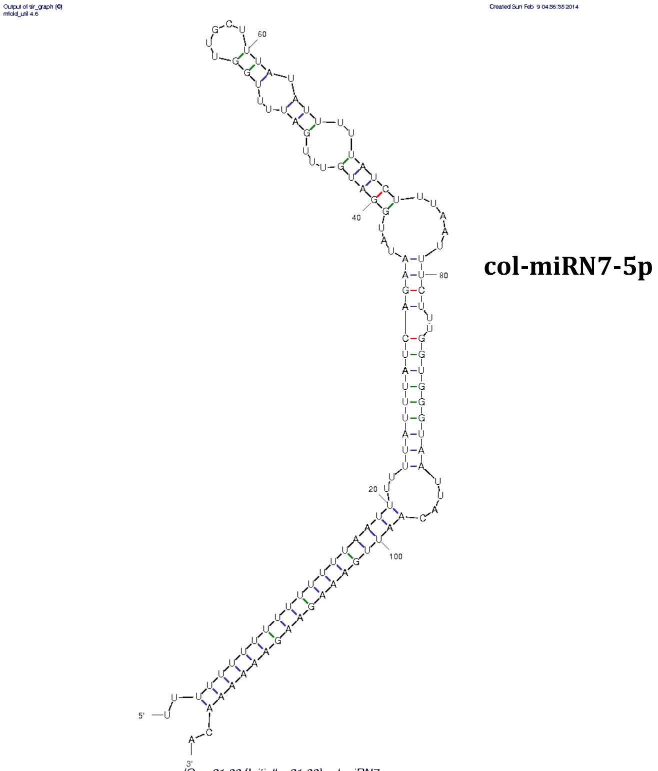

dG = -21.00 [Initially -21.00] col-miRN7

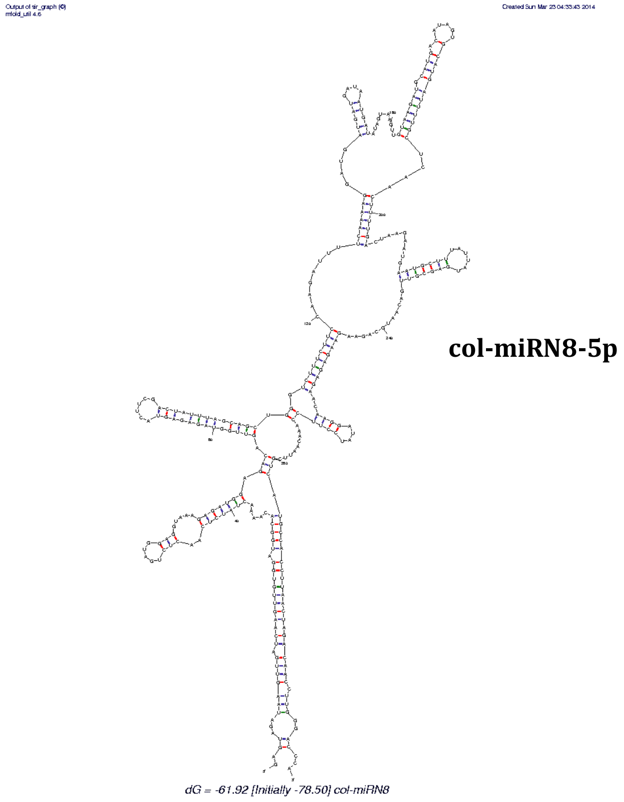

dG = -61.92 [Initially -78.50] col-miRN8

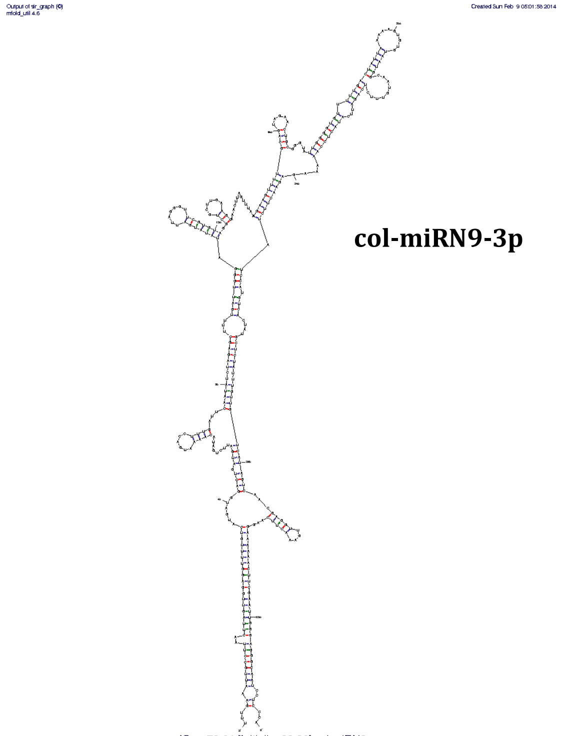

dG = -79.04 [Initially -88.60] col-miRN9

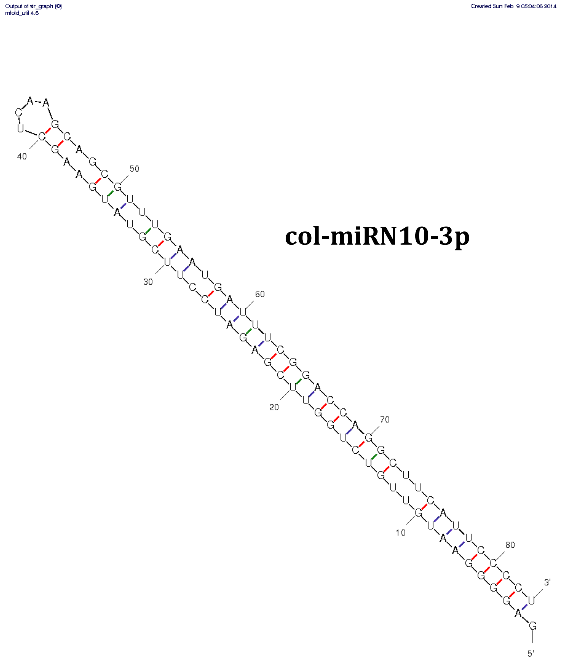

dG = -48.30 [Initially -48.30] col-miRN10

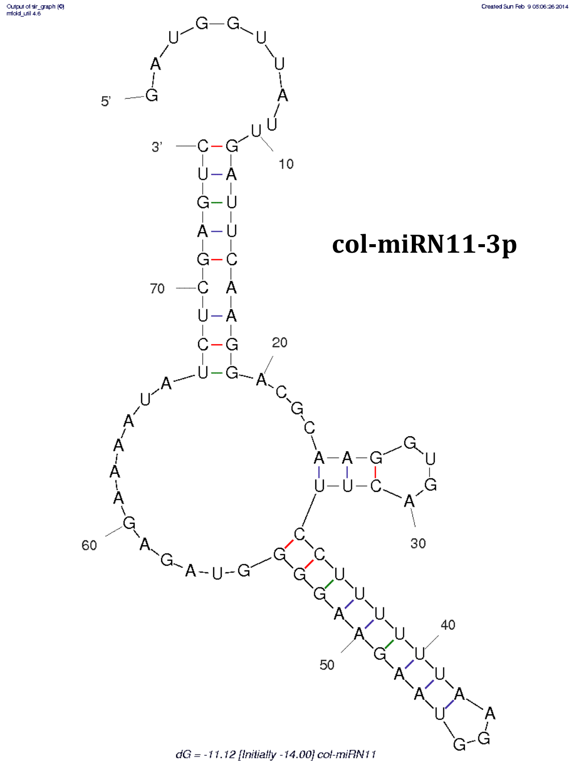

dG = -11.12 [Initially -14.00] col-miRN11

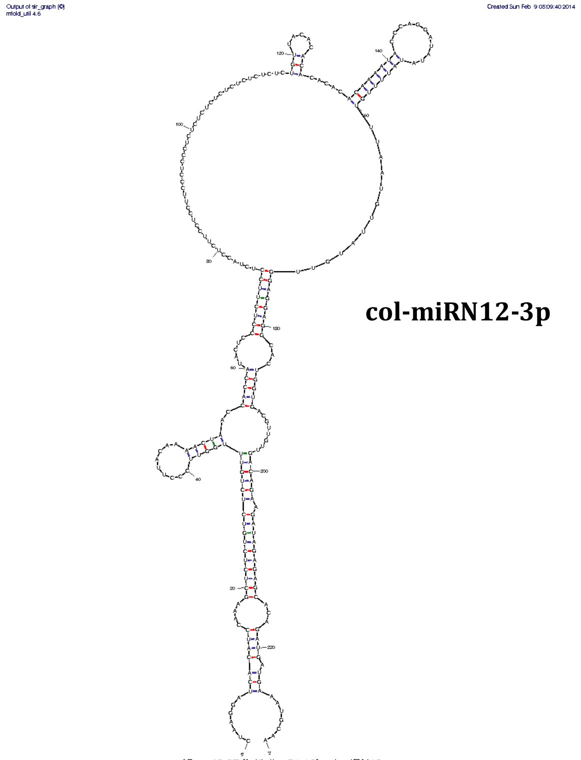

dG = -43.27 [Initially -51.10] col-miRN12

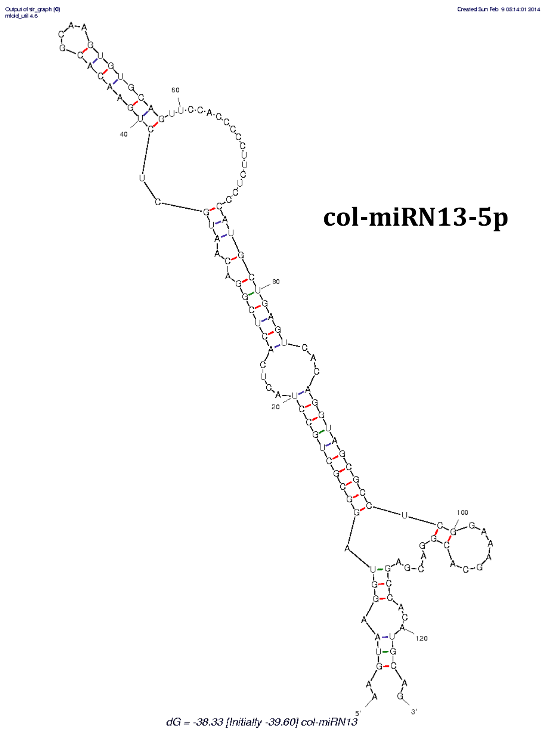
col-miRN13-5p
dG = -38.33 [Initially -39.60] col-miRN13

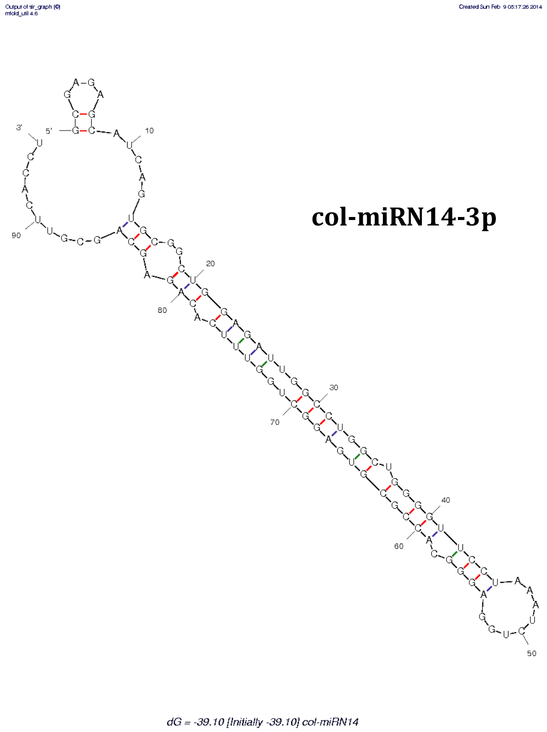
col-miRN14-3p
dG = -39.10 [Initially -39.10] col-miRN14

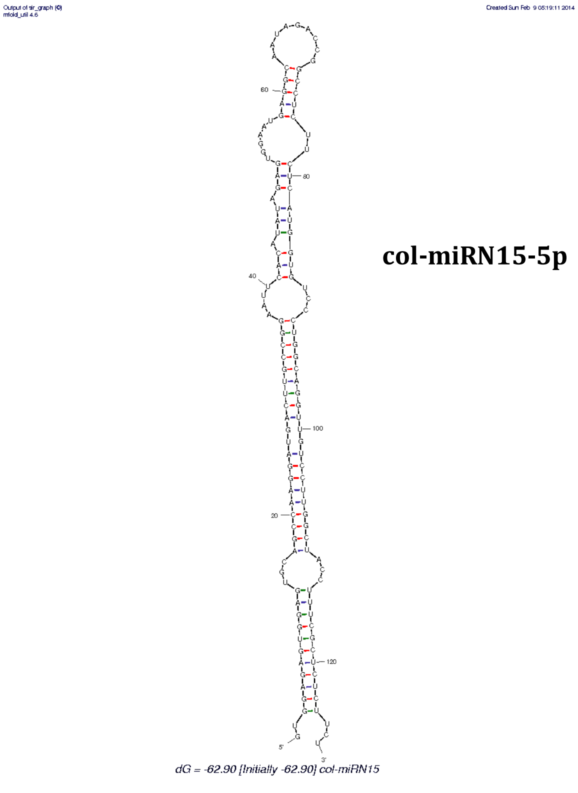
col-miRN15-5p
dG = -62.90 [Initially -62.90] col-miRN15

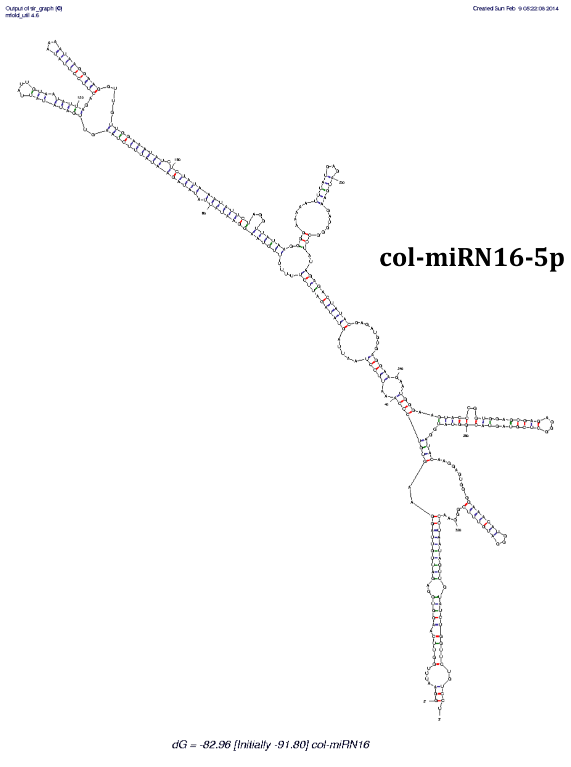
col-miRN16-5p
dG = -82.96 [Initially -91.80] col-miRN16

Output of sir_graph (©)
mfold_util 4.6

Created Sun Feb 9 05:24:28 2014

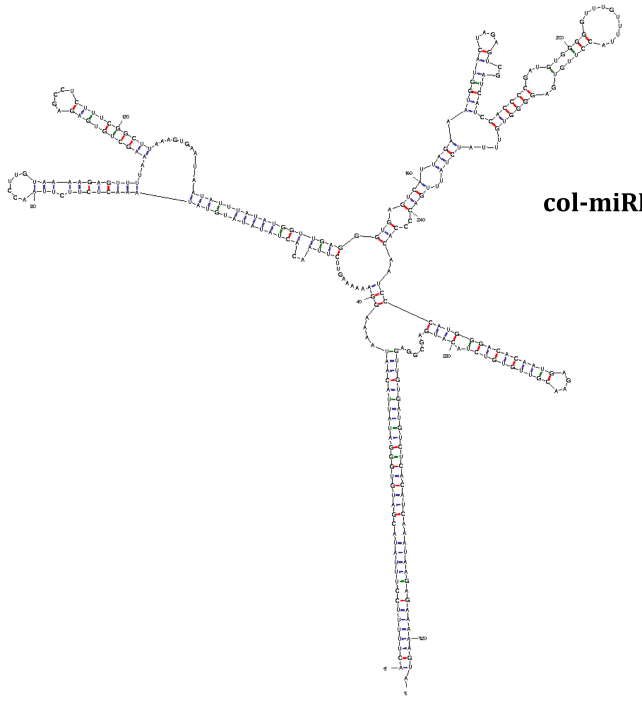

col-miRN17-5p

dG = -97.01 [Initially -104.50] col-miRN17
