# Supplementary material for: High-Throughput Sequencing Reveals Diverse Sets of Conserved, Nonconserved, and Species-Specific miRNAs in Jute
Source: Int J Genomics. 2015 Mar 16;2015:125048. doi: 10.1155/2015/125048 (PMC4378336; doi:10.1155/2015/125048)
Supplement: Supplementary file 1 — Supplementary file-1 contains total and unique reads of different RNAs that have been sequenced. Supplementary file-2 contains all the known miRNAs in jute seedling that have been found in this study. Supplementary file-3 covers the secondary structures of novel miRNA in jute predicted using Mfold. Supplementary file-4 holds the detail of novel jute miRNAs. Supplementary file-5 contains the detail of predicted targets for some of the known miRNAs. Supplementary file-6 covers the detail of predicted targets for novel miRNAs. Supplementary file-7 contains the biological functions of predicted targets. Supplementary file-8 contains the detail of pathways for predicted miRNAs. Supplementary file-9 contains the validation of predicted targets by another tool. [file 125048.f1.zip › Supplementary-3.pdf]

Supplementary file-1:

Type	Unique reads	Total reads
**Exon_antisense**	370	2415
**Exon_sense**	488	612
**Intron_antisense**	1010	8243
**Intron_sense**	1148	19000
**miRNA**	33433	8994892
**rRNA**	53576	538492
**repeat**	1670	76241
**snRNA**	1270	2803
**snoRNA**	673	1378
**tRNA**	12796	891381
**Unannotated tags**	3010673	6108867
